# Deciphering the Molecular Dialogue: Mitochondria, Epigenetics, and Extracellular Vesicles in Placental Function and Pregnancy Complications

**DOI:** 10.1002/cph4.70040

**Published:** 2025-08-18

**Authors:** Yu‐Chin Lien, Rebecca A. Simmons

**Affiliations:** ^1^ Division of Neonatology, Department of Pediatrics Children's Hospital of Philadelphia Philadelphia Pennsylvania USA; ^2^ Center for Women's Health and Reproductive Medicine Perelman School of Medicine, University of Pennsylvania Philadelphia Pennsylvania USA

## Abstract

Placental dysfunction is implicated in the pathogenesis of multiple pregnancy complications. Mitochondria are the powerhouse of the cell and are critical for placental metabolism and function. Several pregnancy complications are associated with oxidative stress and mitochondrial alterations. Mitochondrial function is also essential for epigenetic modifications, which are pivotal in regulating gene expression during pregnancy. Extracellular vesicles (EVs) carry and transfer a variety of biological molecules, including intact mitochondria and mitochondrial components, and act as modifiers of epigenetics in recipient cells. Changes in the EV profile may serve as biomarkers for pregnancy complications. In the present review, we summarize the associations of mitochondrial dysfunction, epigenetic alterations, and changes in EVs that are associated with pregnancy complications. We also describe the link between mitochondria and epigenetics, mitochondria in EVs, and EVs in epigenetic modifications, which provide insight into the possible implications of crosstalk among mitochondria, epigenetics, and EVs in regulating placental function and adverse pregnancy outcomes.

## Graphical abstract

Mitochondria, epigenetic modifications, and extracellular vesicles (EVs) play a pivotal role in regulating placental function. This review explores the associations between mitochondrial dysfunction, epigenetic alterations, and EV changes with pregnancy complications, as well as their interplay in influencing placental function and adverse pregnancy outcomes.

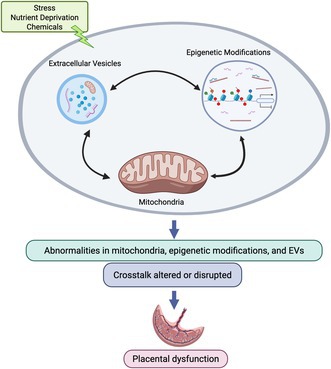

## 1 Introduction

Interorgan communication through a variety of mechanisms, including hormonal signals, cytokines, and extracellular vesicles (EVs), is essential for maintaining physiological homeostasis and response to various challenges. Dysregulation of these communication pathways is implicated in several diseases, including metabolic disorders, systemic inflammation, and cancer (Priest and Tontonoz 2019; Zhao et al. 2023; Argilés et al. 2018). Interorgan communication during pregnancy is vital for maintaining maternal and fetal health and ensuring proper development. This complex interaction involves metabolic, hormonal, and immunological signaling between the mother, placenta, and fetus. The placenta serves as the main communication organ between the mother and fetus during gestation, playing a crucial role in the exchange of gases, nutrients, and fetal waste. Additionally, it produces a variety of hormones and factors that affect maternal physiology and fetal development. However, the mechanisms by which the placenta influences fetal development are not entirely understood. While placental hormones move through maternal and fetal blood vessels, smaller molecules from the placenta require protection from breakdown by both the fetus and mother. These biomolecules include RNAs, peptides, lipids, and mitochondria. They are thought to be transferred to maternal and fetal systems through protective EVs. Placental EVs, particularly their miRNA content, are known to significantly influence maternal systems, including immune, cardiovascular, and reproductive functions. However, there is limited information on how other cargo such as mitochondrial components affect fetal cells and tissues. This review aims to shed light on the role of EVs as a crucial link between maternal and fetal systems, the effects of maternal pathologies on EV cargo, and how understanding biomolecular changes within EVs in both health and disease could help design early diagnostic and intervention strategies to prevent gestational diseases and potential disorders in offspring. The aim of this review is to summarize the main evidence underlying our current understanding of the relationship between mitochondrial dysfunction in the placenta, epigenetic modifications, and EVs in pregnancy complications. We also discuss their interplay and crosstalk, which may provide more comprehensive insight to support future research in developing preventative treatments for these complications.

## 2 Placental Function and Adverse Pregnancy Outcomes

Emerging evidence suggests that placental dysfunction is associated with multiple pregnancy complications such as preeclampsia (PE), fetal growth restriction (FGR), preterm birth (PTB), and gestational diabetes (GDM). Inadequate spiral artery remodeling and trophoblastic invasion, which lead to poor placental perfusion and hypoxia, play important roles in the pathogenesis of PE and FGR (Lyall et al. 2001; Kaufmann et al. 2003). Imbalance between reactive oxygen species (ROS) and antioxidants increases oxidative stress in PE placentas, triggering placental inflammation, and endothelial dysfunction (Siddiqui et al. 2010; Baker et al. 2021; Guerby et al. 2021). Abnormal trophoblast functions are also observed in PE placentas, including altered endocrine and metabolic function, accelerated aging, and increased cell death (Chen et al. 2012; Mayne et al. 2017; Cali et al. 2013). Placental insufficiency is also associated with a significant proportion of preterm births, especially early preterm births (Kourtis et al. 2014; Morgan 2016). An imbalance between substrate supply and metabolic demands in the placenta, as evidenced by altered energy metabolism and mitochondrial function, is observed in placentas from spontaneous PTB (Elshenawy et al. 2020; Lien et al. 2021, 2025). Placental dysfunction is also a contributor to the development of GDM. Dysregulation of placenta‐derived hormones, such as human placental lactogen, estrogen, progesterone, leptin, and cortisol, can exacerbate maternal insulin resistance and increase the risk of GDM (Jones 2001; Lain and Catalano 2007).

## 3 Mitochondria and Pregnancy Complications

The placenta has a high metabolic rate, and maternal nutrients are not only transferred to the fetus but also used to meet energy requirements of the placenta. Mitochondria are the powerhouse of the cell and play critical roles in energy production, nutrient metabolism, redox homeostasis, and oxidative stress, inflammation, and cell death. In the placenta, mitochondria generate ATP through oxidative phosphorylation and maintain cellular redox homeostasis. They also play a role in the synthesis of steroid hormones critical for maintaining pregnancy, such as progesterone, in the syncytiotrophoblast (Martinez et al. 2015). Thus, mitochondria play a central role in regulating placental function and healthy pregnancy outcomes.

Mitochondrial dysfunction in the placenta is increasingly recognized as a critical factor in the pathophysiology of multiple pregnancy complications. Disruptions in placental metabolism, particularly mitochondrial fatty acid oxidation, and altered expression of mitochondrial proteins regulating the respiratory chain, fatty acid oxidation, steroid hormone production, inflammation, autophagy, and apoptosis were observed in the placentas from women with spontaneous PTB, PE, or GDM (Elshenawy et al. 2020; Lien et al. 2021, 2025; Shi et al. 2013; Zhou et al. 2017; Berkane et al. 2018; Muralimanoharan et al. 2016). Abnormal mitochondrial dynamics, ultrastructure, biogenesis, and mitochondrial DNA (mtDNA) copy numbers in the placenta are associated with PE, FGR, and GDM (Shi et al. 2013; Zhou et al. 2017; Berkane et al. 2018; Naha et al. 2020; Poidatz et al. 2015; Kolac et al. 2021; Meng et al. 2015). Increased oxidative stress, along with increased lipid peroxidation and nitrosative damage, and compromised mitochondrial function and antioxidant enzymes, including catalase and glutathione levels, have been reported in placentas from PE, FGR, PTB, and GDM (Yang et al. 2021; Kaur et al. 2008; Muralimanoharan et al. 2012; Yung et al. 2019; Biri et al. 2007, 2006; Singh et al. 2023; Ahamed et al. 2009; Sarina et al. 2019; Ramírez‐Emiliano et al. 2017; Fisher et al. 2021). Taken together, these studies demonstrate that mitochondrial dysfunction underlies many pregnancy complications.

## 4 Placental Epigenetic Modifications and Pregnancy Complications

**FIGURE 1:**
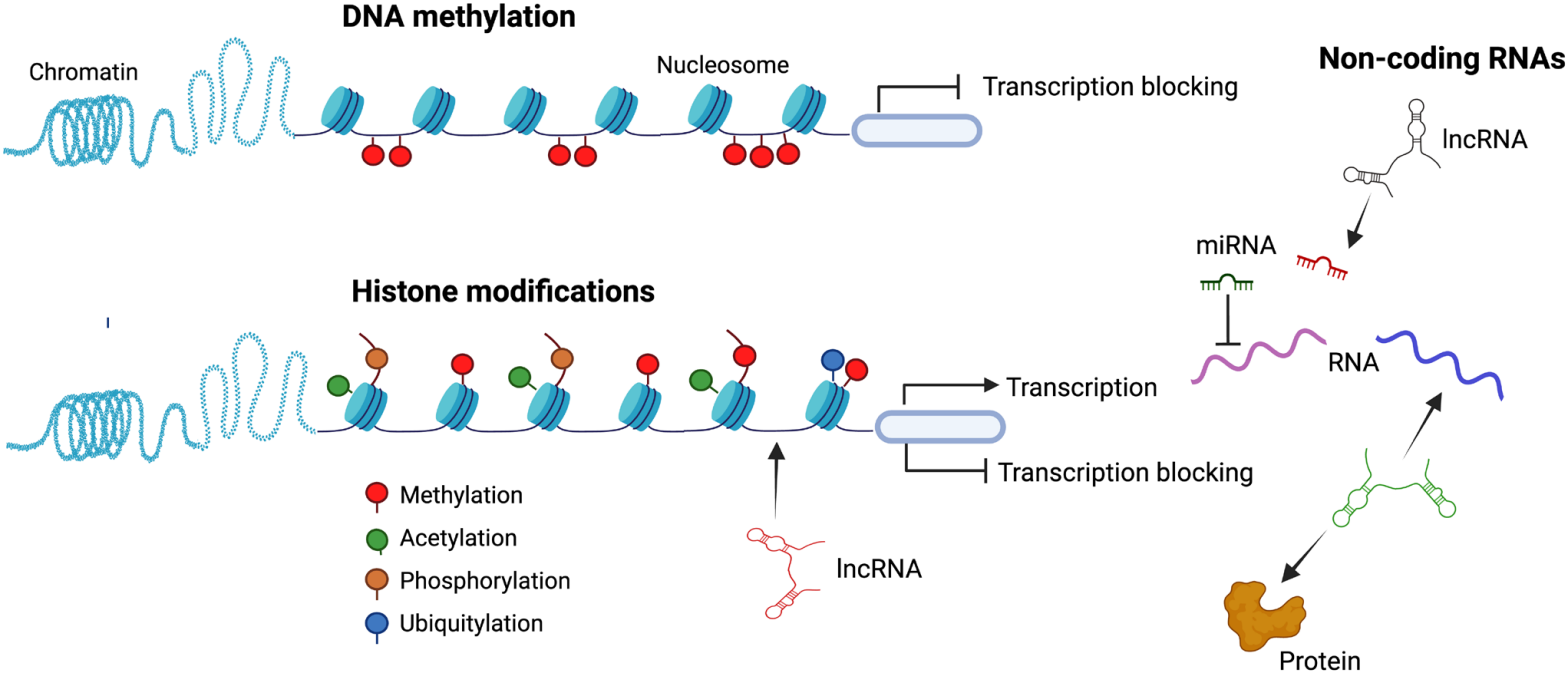
Epigenetic modifications regulate gene expression through multiple mechanisms. DNA methylation at CpG dinucleotides typically leads to gene silencing. The most common histone modifications include methylation, acetylation, phosphorylation, and ubiquitination. Generally, histone acetylation or phosphorylation enhances gene expression, whereas histone methylation and ubiquitination can either activate or repress gene expression, depending on the specific histone protein being modified. Additionally, non‐coding RNAs play a crucial role in gene expression. MicroRNAs (miRNAs) bind to specific sequences in the 3′ untranslated region of target mRNAs, negatively regulating gene expression post‐transcriptionally. Long non‐coding RNAs (lncRNAs) modulate gene expression by interacting with DNA, RNA, and proteins.

Epigenetic changes, including DNA methylation, histone modifications, and non‐coding RNAs, regulate gene expression without alterations in the DNA sequence (Figure 1). Epigenetic modifications in the placenta are pivotal for lineage specification in the preimplantation placenta, influencing gene expression patterns that are vital for proper fetal and placental development, and potentially impacting maternal and fetal health outcomes. Environmental exposures, including maternal diet, stress, and toxicants, are also associated with epigenetic changes in the placenta and may predispose the fetus to long‐term adverse health outcomes such as metabolic disorders, cardiovascular disease, and cognitive function (Tekola‐Ayele et al. 2020; Diez‐Ahijado et al. 2024; Kumar et al. 2024; Strakovsky and Schantz 2018).

Epigenetic changes have been observed in placentas from various pregnancy complications. Multiple studies have reported global DNA methylation changes in PE placentas that are associated with altered expression of genes important for placental function, including TGF‐beta signaling, oxidative phosphorylation, metabolic pathways, and cytokine‐cytokine receptor interaction (Blair et al. 2013; Ching et al. 2014; Kulkarni et al. 2011; Leavey et al. 2018). Hypermethylation of the imprinting gene *H19* in placentas is associated with decreased *H19* expression, which may have an impact on trophoblast invasion and contribute to the pathophysiology of early‐onset PE (Gao et al. 2011; Adam et al. 1996). Placental DNA methylation changes in genes regulating metabolism, immune response, and steroid hormone biosynthesis, particularly *CYP11A1*, are associated with FGR (Shi et al. 2023). In spontaneous PTB placentas, DNA methylation changes have been shown to map to genes regulating inflammation and metabolism, such as *IGF1R* and *PPARGC1A* (Wang et al. 2022). However, whether these DNA methylation changes are associated with gene expression changes has not been determined in this study. In placentas from women with GDM, DNA methylation in genes regulating insulin resistance and metabolic homeostasis is altered (Alexander et al. 2018; Lu et al. 2022; Lesseur et al. 2014; Houde et al. 2015; El Hajj et al. 2013).

Alterations in histone modifications in placentas from pregnancy complications are also reported. H3K4me3 and H3K9ac histone modifications are reduced, and global levels of H3K27ac are increased in PE placentas (Meister et al. 2021; Zhang, Kim, et al. 2021). These histone modification changes may lead to abnormal expression of genes critical for trophoblast and endothelial functions, including vascular endothelial growth factor (*VEGF*), placental growth factor (*PGF*), endoglin (*ENG*), and Fms‐like tyrosine kinase‐1 (*FLT1*) (Meister et al. 2021; Zhang, Kim, et al. 2021). In placentas from women with GDM, decreased levels of H3K9ac and H3K27ac histone modifications have been reported (Hepp et al. 2018; Jiang et al. 2020).

Moreover, changes in non‐coding RNAs in placentas are associated with pregnancy complications. The expression of multiple miRNAs is reported dysregulated in PE placentas (Pineles et al. 2007; Zhu et al. 2009). Changes in miRNA levels in human preterm placenta are also associated with inflammation and preterm labor (Sun et al. 2018). Further, lnc‐SNHG29 is increased in human spontaneous preterm placenta, and these changes were linked to altered trophoblast cell senescence and inflammation in in vitro studies (Jiang et al. 2021). Dysregulation of miRNAs, lncRNAs, and circRNAs in GDM placentas has also been reported in multiple studies and were associated with abnormal placental metabolism and function (Du et al. 2021). These are intriguing studies; however, to date, no study has definitively demonstrated that changes in epigenetic modifications are causal to adverse pregnancy outcomes in the human.

## 5 Mitochondrial‐to‐Nuclear Communication and Epigenetics

**FIGURE 2:**
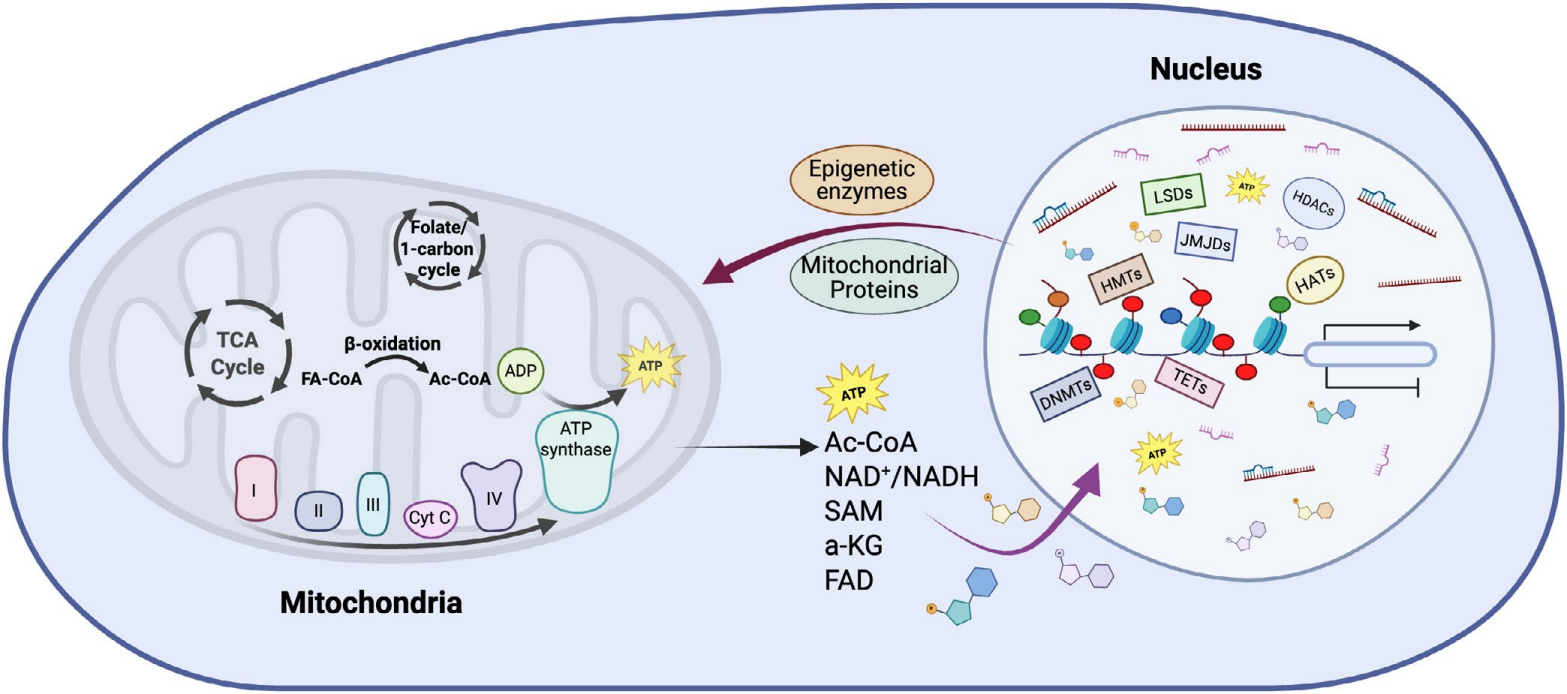
Mitochondrial function and epigenetic modifications. Mitochondria provide the energy and intermediate metabolites necessary for generating epigenetic marks, such as histone acetylation, histone methylation, and DNA methylation. Conversely, epigenetic modifications and enzymes regulate mitochondrial biogenesis and function. Ac‐CoA, acetyl‐CoA; a‐KG, a‐ketoglutarate; DNMT, DNA methyltransferase; FA‐CoA, fatty acyl CoA; FAD, flavin adenine dinucleotide; HAT, histone acetyltransferase; HDAC, histone deacetylase; HMT, histone methyltransferase; JMJD, jumonji C domain demethylase; LSD, Lys‐specific demethylase; SAM, S‐adenosyl methionine; TCA cycle, tricarboxylic acid cycle; TET, ten‐eleven translocation enzyme.

Mitochondrial function is essential in regulating epigenetic modifications. Conversely, epigenetic modifications also modulate mitochondrial protein expression and function (Figure 2). Mitochondria provide the energy and intermediate metabolites necessary for generating epigenetic marks. Acetyl‐CoA, a cofactor generated from several mitochondrial metabolic pathways, provides an acetyl group to histone acetyltransferases for lysine residual acetylation (McCullough and Marmorstein 2016). In contrast, Sirtuins, the Class III histone deacetylases (HDACs), require NAD^+^ as a cofactor to remove acetyl groups (Seto and Yoshida 2014). NAD^+^/NADH levels and homeostasis are tightly linked to mitochondrial function (Nasuhidehnavi et al. 2024). S‐adenosyl methionine (SAM), whose generation depends on the folate cycle, ATP, and mitochondrial metabolism, is needed for both histone and DNA methylation (Ducker and Rabinowitz 2017). Histone methyltransferases transfer methyl groups from SAM to lysine or arginine residues on histone proteins (Teperino et al. 2010). DNA methyltransferases (DNMTs) also use SAM as a methyl donor for generating 5‐methylcytosine (5mC) (Robertson 2005). As for histone demethylation, lysine‐specific demethylases (LSDs) depend on flavin adenine dinucleotide (FAD) (Song et al. 2023; Kooistra and Helin 2012), and jumonji C domain demethylases (JMJDs) use α‐ketoglutarate (α‐KG) as a cofactor (Kooistra and Helin 2012; Xiao et al. 2012). Similarly, ten‐eleven translocation (TET) enzymes are responsible for oxidizing 5mC to 5hmC, triggering a DNA demethylation process, and they also use α‐KG as a cofactor (Xu et al. 2011). The activity of JMJDs and TETs can be inhibited by succinate and fumarate, and the levels of FAD, α‐KG, succinate, and fumarate are tightly regulated by the TCA cycle and mitochondrial function (Martínez‐Reyes and Chandel 2020). In summary, mitochondrial function is essential for epigenetic regulation, and it provides the energy and cofactors necessary for the activity of multiple enzymes involved in DNA methylation and histone modifications.

Multiple epigenetic enzymes are shown to regulate mitochondrial biogenesis and function. For example, LSD1 plays an important role in regulating mitochondrial gene expression and modulating mitochondrial respiration, fatty acid oxidation, and glycolysis (Hino et al. 2012; Duteil et al. 2016, 2014; Sakamoto et al. 2015). Inhibition of LSD1 function by siRNA or selective inhibitors in adipocytes induces expression of PPARγ coactivator‐1α (*PPARGC1A*), pyruvate dehydrogenase kinase 4 (*PDK4*), and fatty acid transporter 1 (*FATP1*), resulting in the activation of mitochondrial respiration (Hino et al. 2012). KDM5A, a histone demethylase in JMJD family, regulates expression of genes required for mitochondrial biogenesis and structure (Váraljai et al. 2015; Liu and Secombe 2015). In vitro studies show that silencing the lysine methyltransferase SETD7 (SET7/9) upregulates the expression of antioxidant enzymes superoxide dismutase 2 (*SOD2*) and catalase (*CAT*), enhancing ROS clearance and improving mitochondrial function through upregulation of *PPARGC1A* (He et al. 2015). DNA methylation also regulates the gene expression of multiple mitochondrial proteins, including uncoupling protein‐1 (*UCP1*), carnitine palmitoyltransferase 1A (*CPT1A*), *PPARGC1A*, and *PDK4* (Shore et al. 2010; Kiskinis et al. 2007; Irvin et al. 2014; Barrès et al. 2009, 2012). Furthermore, DNMT1, TET1, and TET2 are observed in mitochondria, suggesting methylation/demethylation of mtDNA as an epigenetic mechanism in regulating mtDNA transcription (Shock et al. 2011; Dzitoyeva et al. 2012). These studies suggest that epigenetic modifications play a critical role in regulating mitochondrial gene expression and function; however, it remains to be determined whether these pathways regulate mitochondrial function in placentas.

## 6 Extracellular Vesicles and Pregnancy Complications

Extracellular vesicles are nanometer‐sized, cell‐derived membrane‐bound small vesicles which carry a variety of biological molecules, including intact mitochondria, mitochondrial components, proteins, lipids, DNA fragments, coding and non‐coding RNAs, and metabolites (Doyle and Wang 2019; Zhang, Liang, et al. 2023). The release of EVs is an active cellular process involving EV biogenesis, trafficking, and secretion and is energy dependent. EVs facilitate intercellular communication and play a pivotal role in normal pregnancy as well as in pregnancy complications. During pregnancy, EVs are released from the placenta, enter the maternal bloodstream, and promote maternal adaptation, immune tolerance, trophoblast invasion, and spiral artery remodeling (Sarker et al. 2014; Bai et al. 2021; Tannetta et al. 2013). In maternal plasma, the number of EVs containing the placenta‐specific marker placenta alkaline phosphatase (PLAP) increases progressively with gestational age at early pregnancy (Sarker et al. 2014). EVs derived from first trimester placenta carry immunomodulatory molecules, such as B7H‐1 (CD274), B7‐H3 (CD276), and HLA‐G5, and may modulate the maternal immunological environment (Kshirsagar et al. 2012). Further, NK group 2 member D (NKG2D) ligand expressing EVs from the placenta can downregulate NKG2D in Natural Killer (NK), CD8(+), and gamma‐delta T cells and impair their cytotoxicity leading to immune tolerance (Hedlund et al. 2009).

Alterations in EV concentration are associated with multiple pregnancy complications. While the levels of total plasma EVs and EVs derived from the placenta are elevated in patients with preeclampsia (Tannetta et al. 2013; Knight et al. 1998; Salomon et al. 2017; Pillay et al. 2019) and GDM (Salomon et al. 2016; James‐Allan et al. 2020; Franzago et al. 2021), plasma EVs are significantly lower in women giving birth to children with FGR (Miranda et al. 2018; Klemetti et al. 2024). However, accurate quantification of EVs is difficult as standard methods enumerate not only EVs, but also other similarly sized non‐EV particles. Thus, it is not clear if these changes in EV numbers reflect altered EV biology. There is a critical need to develop improved techniques for EV isolation and quantification. However, importantly, EV cargo is altered in several pregnancy complications. For example, multiple proteins in EVs are altered in women with PE, including higher levels of PE biomarkers Eng, soluble Eng (sEng), Flt‐1, soluble Flt‐1 (sFlt‐1), and PLAP, and lower levels of Syncytin‐1 (Tannetta et al. 2013; Chaparro et al. 2016; Chang et al. 2018; Rajakumar et al. 2012; Levine et al. 2020). Eng and Flt‐1/sFlt‐1 in EVs can bind to their ligands, such as VEGF, PGF, and TGF‐β, in vitro (Tannetta et al. 2013). sEng and sFlt‐1 in EVs from women with PE are horizontally transferred to human umbilical vein endothelial cells in culture and can attenuate their proliferation, migration, and tube formation (Chang et al. 2018). Thus, EV‐associated Eng and Flt‐1/sFlt‐1 could contribute to the increased circulating levels measured in PE patients and the perturbation of maternal vascular endothelium (Tannetta et al. 2013). Several studies also report significant changes in the content of miRNAs critical for trophoblast and placental function in EVs from PE patients (Salomon et al. 2017; Biró et al. 2017; Motawi et al. 2018; Shen et al. 2018; Hromadnikova et al. 2019; Pillay et al. 2016; Chan and Loscalzo 2010). Furthermore, changes in EV cargo, including lipids and miRNAs, are associated with FGR (Klemetti et al. 2024; Hromadnikova et al. 2019; Rodosthenous et al. 2017), and alterations in EV miRNAs and proteins involved in inflammatory, metabolic, and coagulation processes are associated with the risk of PTB (Fallen et al. 2018; Menon et al. 2019; McElrath et al. 2019; Ezrin et al. 2015; Cantonwine et al. 2016). Many miRNAs critical in regulating trophoblast proliferation, fatty acid biosynthesis, insulin signaling, and inflammatory response, as well as dipeptidyl peptidase IV (DPPIV), which cleaves glucagon‐like polypeptide‐1 (GLP‐1), are altered in EVs from women with GDM (Kandzija et al. 2019; Herrera‐Van Oostdam et al. 2020; Shah et al. 2021; Nair et al. 2018; Gillet et al. 2019).

Functional studies show that nonpregnant mice infused with EVs from GDM women developed glucose intolerance and had attenuated glucose‐stimulated insulin secretion and muscle basal insulin signaling (James‐Allan et al. 2020). Placental EVs from GDM women can decrease insulin‐stimulated migration and glucose uptake in primary skeletal muscle cells from patients with normal insulin sensitivity (Nair et al. 2018). Adipose tissue‐derived EVs from patients with GDM exhibit changes in the levels of proteins regulating mitochondrial function, sirtuin signaling, mTOR signaling, and oxidative phosphorylation, which may impact glucose metabolism in the placenta (Jayabalan et al. 2019). Moreover, we recently reported that circulating EVs from pregnant women infected with severe acute respiratory syndrome coronavirus 2 (SARS‐CoV‐2) inhibit the invasion of primary human first‐trimester extravillous trophoblasts in vitro (Golden et al. 2025).

Taken together, these studies demonstrate that alterations in EV number and cargo may contribute to the pathogenesis of pregnancy complications and serve as potential biomarkers. Development of improved methods for accurate EV quantification and cargo characterization is urgently needed. In addition, further studies are required to investigate the association between EV alterations and pregnancy complications at early gestational stages. These efforts may facilitate the identification of novel biomarkers, in addition to sEng, Flt‐1, sFlt‐1, for early diagnosis and inform the development of targeted interventions to prevent pregnancy complications and adverse outcomes.

## 7 The Link Between Extracellular Vesicles and Mitochondria

In addition to delivering proteins, lipids, nucleic acids, and metabolites to recipient cells, emerging evidence shows that EVs can also carry intact mitochondria and mitochondrial components, including mtDNA (Jang et al. 2019; Puhm et al. 2019). Moreover, mitochondria‐derived EVs, mitovesicles, have also been identified in EVs isolated from murine and human brains by high‐resolution density gradient (D'Acunzo et al. 2022, 2021). Mitovesicles exhibit a double‐membrane structure and electron density, are enriched in mitochondrial proteins while lacking microvesicle and exosomal markers, and contain a lipid profile consistent with a mitochondrial origin (D'Acunzo et al. 2021). EVs carrying polarized mitochondria, as indicated by MitoTracker Deep‐Red staining, from cultured human brain endothelial cells can transfer mitochondria to the recipient cells (D'Souza et al. 2021). Seahorse analysis indicates that polarized mitochondria enhance mitochondrial function and ATP production in recipient hypoxic endothelial cells in culture, as well as in neurons within acute mouse brain cortical and hippocampal slices. This improvement can promote endothelial cell survival under ischemic conditions (D'Souza et al. 2021).

Mitochondrial components can be transferred to target tissues by EVs and regulate mitochondrial and cellular functions in the recipient cell. EVs from metastatic breast cancer tissue carry the full mitochondrial genome, which can be horizontally transferred to recipient cells in culture (Sansone et al. 2017). Seahorse analysis indicates that mtDNA from metastatic breast cancer‐derived EVs increases oxidative phosphorylation (OXPHOS) capacity in recipient cells with low OXPHOS activity and mtDNA levels (Sansone et al. 2017). Further, EVs from human adipose‐derived mesenchymal stem cells can transfer mitochondrial components to LPS‐challenged alveolar macrophages in culture, leading to the elevation of mtDNA level, mitochondrial membrane potential, OXPHOS activity, and ATP generation, while relieving mitochondrial ROS stress as indicated by decreased levels of mitochondrial superoxide indicator MitoSOX red staining (Xia et al. 2022). Restoring mitochondrial integrity via EV treatment enables alveolar macrophages to shift to an anti‐inflammatory phenotype, as indicated by decreased IL‐1β, TNF‐α, and iNOS secretion and increased anti‐inflammatory cytokines IL‐10 and Arg‐1 (Xia et al. 2022). In contrast, EVs can also transfer damaged mitochondrial components to recipient cells. For example, EVs from antimycin A or oligomycin‐treated, energetic stressed adipocytes carry respiration‐competent but oxidatively damaged mitochondrial particles, which can trigger ROS bursts leading to compensatory antioxidant signaling in recipient cardiomyocytes (Crewe et al. 2021). EVs derived from cultured inflammatory M1 macrophages contain mitochondrial components, including mitochondrial proteins (ATP5A, VDAC, COX IV, PDHA) and mtDNA. These mitochondria in EVs can transfer to pancreatic beta cells in culture, where they fuse with beta cell mitochondria, leading to lipid peroxidation, mitochondrial disruption, and ferroptosis in the setting of acute pancreatitis (Gao et al. 2024). Studies on EVs and mitochondria in the placenta and pregnancy complications are limited. Increased mtDNA in EVs derived from antiphospholipid antibody‐exposed human placental explants activates recipient endothelial cells, which may increase the risk of preeclampsia (Tong et al. 2017). We also recently demonstrated increased mtDNA levels in circulating large EVs from pregnant women infected with SARS‐CoV‐2 (Golden et al. 2025). Future investigations into the alterations of mitochondrial components in EVs during pregnancy complications, as well as direct evidence of their roles in regulating placental function in both normal and complicated pregnancies, are needed and will provide valuable insights in the field.

## 8 The Link Between Extracellular Vesicles and Epigenetic Modifications

EVs also transmit proteins, nucleic acids, and metabolites that participate in epigenetic modifications in recipient cells. In addition to miRNAs, other non‐coding RNAs critical in epigenetic regulation have been identified in EVs, including lncRNA, tRNA, and piRNA (Abramowicz and Story 2020). Circulating EVs from acute coronary syndrome (ACS) patients are enriched with DNMT1, DNMT3A, and DNMT3B mRNA (Schiano et al. 2022). Peripheral blood mononuclear cells from healthy donors treated with ACS‐derived EVs show methylation changes in thousands of CpG sites as well as altered expression of genes important for the onset of several cardiovascular diseases, including *SRC, NOTCH1*, and *FOXO3* (Schiano et al. 2022). However, the direct evidence linking DNA methylation and gene expression changes in peripheral blood mononuclear cells to increased DNMT mRNA levels in ACS‐derived EVs is lacking in this study. Histone modifications are also modulated by EVs. Multiple studies have reported that lysine demethylase 6B (KDM6B), which demethylates H3K27me3, is the target of miRNAs in EVs derived from endothelial progenitor cells and adipose‐derived mesenchymal stem cells (He et al. 2020; Zhang, Liu, et al. 2021; Qi et al. 2021). Silencing of KDM6B by these miRNAs can protect cells from acute injury, including acute kidney injury and brain ischemic injury. The expression of histone deacetylases, such as HDAC1, HDAC2, and HDAC4, and histone acetylation levels in the recipient cells are also regulated by EVs derived from various cell types (Han et al. 2020; Shi et al. 2022; Qu et al. 2021; Zhang, Du, et al. 2023). EVs from human umbilical cord‐derived mesenchymal stem cells contain miR‐410 and can be delivered to neurons in culture, where it negatively regulates *HDAC1* expression, resulting in the upregulation of *EGR2/Bcl2* and neuron survival (Han et al. 2020). EVs from anti‐inflammatory M2 macrophages can transport miR‐23a‐3p to the microglia and decrease ubiquitin‐specific proteinase 5 (USP5) to stabilize HDAC2 via deubiquitination, which leads to the inhibition of *NRF2* expression and pain alleviation (Qu et al. 2021). In these studies, downregulation of HDACs induced by EVs can help prevent hypoxia‐ischemia brain damage, inflammatory pain, or diabetic osteoporosis. Future studies are warranted to elucidate the role of EVs and EV mitochondrial components in modulating placental epigenetic modifications in both normal and complicated pregnancies.

## 9 Conclusions and Perspectives

**FIGURE 3:**
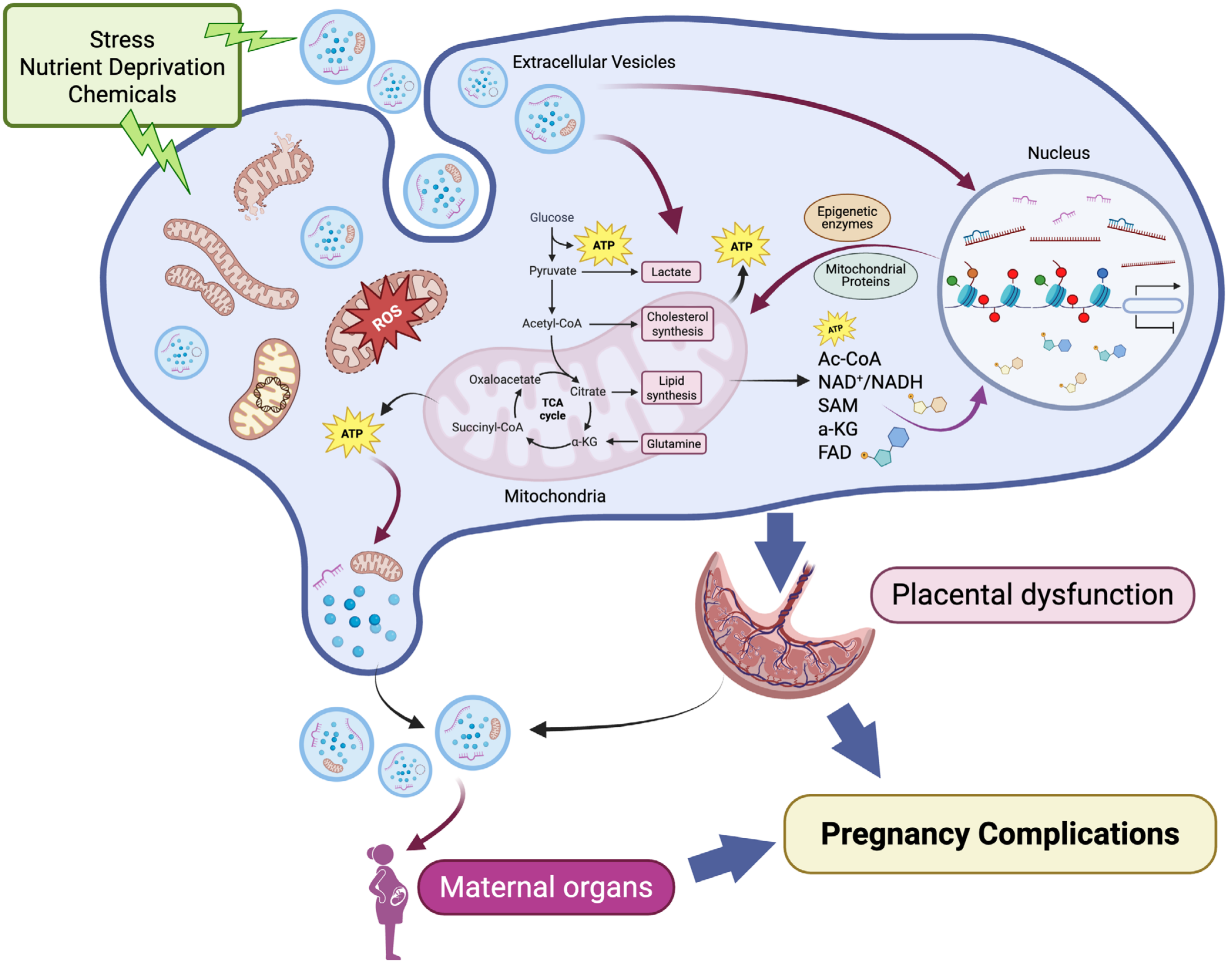
Potential crosstalk and interplay between mitochondria, epigenetics, and EVs in the placenta. Mitochondria provide energy and cofactors needed for epigenetic modifications in the nucleus. Epigenetic modifications regulate the expression of multiple epigenetic enzymes and mitochondrial proteins, which can regulate mitochondrial biogenesis and function. Biogenesis and secretion of EVs require energy. The components of EV cargo can alter mitochondrial function, inducing epigenetic modifications, and changes in gene expression in the recipient cell. Upon environmental stimuli, such as stress, nutrient deprivation, or chemical exposures, mitochondrial function, epigenetic modifications, or EVs concentration and cargo can be altered, which may lead to placental dysfunction. A dysfunctional placenta may further release abnormal quantities of EVs or EVs with altered cargo, potentially affecting maternal and fetal systems and organs, ultimately resulting in pregnancy complications and adverse fetal outcomes.

In conclusion, multiple studies demonstrate that EVs carry mitochondria cargo which can be transmitted to the recipient cell, resulting in altered metabolic function. Emerging data suggest that EV cargo can also induce epigenetic modifications, which in turn modulate gene expression. Further, EV concentration and cargo are altered in pregnancy complications and may serve as important biomarkers. Thus, we propose that the crosstalk and interplay among EVs, mitochondria, and epigenetics may play critical roles in regulating placental function and pregnancy outcomes (Figure 3).

While existing studies highlight the link between EVs, mitochondria, and epigenetics, direct evidence of their crosstalk in the placenta during healthy pregnancy or complications remains to be established. Moreover, the origins of EVs critical for maternal‐placental‐fetal communication and placental function modulation during pregnancy, the detailed mechanisms underlying the interplay between EVs, mitochondria, and epigenetics in placentas, and how their crosstalk influences fetal development, as well as specific placental cell types affected in various pregnancy complications, require further investigation. Developing appropriate animal models that mimic human pregnancy complications, along with advanced in vitro models such as the placenta‐on‐a‐chip system, placenta explants, or organoids, as well as innovative tools for precise EV cargo characterization, will significantly advance research in this field. Further, race and social determinants of health that differentially impact pregnancy outcomes across racial groups play important roles in pregnancy complications that frequently are attributed to placental dysfunction. In the United States, the overall preterm birth rate is 52% higher for Black women than for White women (Burris et al. 2019). The incidence of other pregnancy complications, such as preeclampsia, is also higher among Black women (Boakye et al. 2021). We also observed greater dysfunction and inflammation in Black compared to White spontaneous PTB placentas (Lien et al. 2025). Women from lower‐resource communities may have poor nutrition or higher exposure to stress and environmental chemicals, which can alter mitochondrial function, epigenetic modifications, and EV profiles (cargo, size distribution, and numbers) (Schuster et al. 2019; Santos et al. 2019; Noren Hooten et al. 2022). It is critically important to further investigate whether social determinants and changes in mitochondrial function, epigenetics, and the EV profile underlie placental dysfunction and race differences in the prevalence of pregnancy complications. Overall, this review serves as a basis for future evidence‐based hypothesis testing as the research field linking pregnancy complications and mitochondria, epigenetics, and EVs continues to grow, as well as provides more comprehensive insight to support future research in developing preventative treatments for these complications.

## Author Contributions

**Yu‐Chin Lien:** writing – original draft preparation. **Rebecca A. Simmons:** writing – review and editing. Both authors have read and agreed to the published version of the manuscript.

## Conflicts of Interest

The authors declare no conflicts of interest.

## Data Availability

The authors have nothing to report.
